# Pre-operative right ventricular echocardiographic parameters associated with short-term outcomes and long-term mortality after CABG

**DOI:** 10.1530/ERP-18-0041

**Published:** 2018-11-19

**Authors:** Mohammed Andaleeb Chowdhury, Jered M Cook, George V Moukarbel, Sana Ashtiani, Thomas A Schwann, Mark R Bonnell, Christopher J Cooper, Samer J Khouri

**Affiliations:** 1 Division of Cardiovascular Medicine, University of Toledo Medical Center, Toledo, Ohio, USA; 2 University of Toledo Medical Center, Toledo, Ohio, USA; 3 Division of Cardiothoracic Surgery, University of Toledo Medical Center, Toledo, Ohio, USA

**Keywords:** echocardiography, right ventricle dysfunction, CABG, post-operative complications

## Abstract

**Background:**

This analysis aims to assess the prognostic value of pre-operative right ventricular echocardiographic parameters in predicting short-term adverse outcomes and long-term mortality after coronary artery bypass graft (CABG).

**Methods:**

Study design: Observational retrospective cohort. Pre-operative echocardiographic data, perioperative adverse outcomes (POAO) and long-term mortality were retrospectively analyzed in 491 patients who underwent isolated CABG at a single academic center between 2006 and 2014.

**Results:**

Average age of enrolled subjects was 66 ± 11.5 years with majority being male (69%). 227/491 patients had 30 days POAO (46%); most common being post-operative atrial fibrillation (27.3%) followed by prolonged ventilation duration (12.7%). On multivariate analysis, left atrial volume index ≥42 mL/m^2^ (LAVI) (OR (95% CI): 1.98 (1.03–3.82), *P* = 0.04), mitral E/A >2 (1.97 (1.02–3.78), *P* = 0.04), right atrial size >18 cm^2^ (1.86 (1.14–3.05), *P* = 0.01), tricuspid annular plane systolic excursion (TAPSE) <16 mm (1.8 (1.03–3.17), *P* = 0.04), right ventricular systolic pressure (RVSP) ≥36 mmHg (pulmonary hypertension) (1.6 (1.03–2.38), *P* = 0.04) and right ventricle myocardial performance index (RVMPI) >0.55 (1.58 (1.01–2.46), *P* = 0.04) were found to be associated with increased 30-day POAO. On 3.5-year follow-up, cumulative survival was decreased in patients with myocardial performance index (MPI) ≥0.55 (log rank: 4.5, *P* = 0.034) and in patients with mitral valve E/e′ ≥14 (log rank: 4.9, *P* = 0.026).

**Conclusion:**

Pre-operative right ventricle dysfunction (RVD) is associated with increased perioperative complications. Furthermore, pre-operative RVD and increased left atrial pressures are associated with long-term mortality post CABG.

## Introduction

Coronary artery bypass graft (CABG) is the most common cardiac surgical procedure in the United States with 1 CABG being performed per 1000 individuals (1). Majority of evidence regarding post-operative outcomes have largely been based on left ventricular (LV) function (2, 3) with a limited number of studies evaluating the role of right ventricle (RV), as its complex geometric shape makes functional assessment challenging. With the introduction of echocardiographic indices of right ventricular function, there has been an increased interest in this subject and currently there is growing evidence that the RV may have a more important role in the overall cardiac function and outcomes than previously appreciated.

Patients with advanced coronary artery disease, LV systolic and diastolic dysfunction often develop some degree of pulmonary hypertension (PH) and RVD. Left heart pathology is responsible for 65–80% of cases of PH (4) and 44% of patients with coronary artery disease have PH (5). During surgery, these patients are at an increased risk for post-operative complications due to acute-onset myocardial dysfunction and PH (6). A compromised RV might not be able to tolerate the sudden rise in pulmonary pressures leading to post-operative RV and circulatory failure (7). Therefore, pre-operative RVD could potentially be an important marker for post CABG adverse outcomes, but to date has not been included in either short-term or long-term mortality risk models.

The importance of RV systolic and diastolic echocardiographic parameters as a prognostic tool in patients with symptomatic heart failure has already been established (8). Identifying pre-operative RVD by echocardiography offers a relatively easy, non-invasive and effective strategy to recognize high-risk patients (9). Recent studies have shown that echocardiographic parameters of RV function and PH were predictive of perioperative mortality in patients undergoing cardiac valve surgeries (10, 11, 12). However, data on RV echocardiographic indices and post CABG outcomes are limited.

In the current study, we tested the hypothesis that pre-operative RVD is associated with adverse post CABG outcomes. Specifically, we evaluate whether parameters of RVD were associated with post-operative complications and long-term mortality after CABG.

## Methods

### Study population

**Figure 1:**
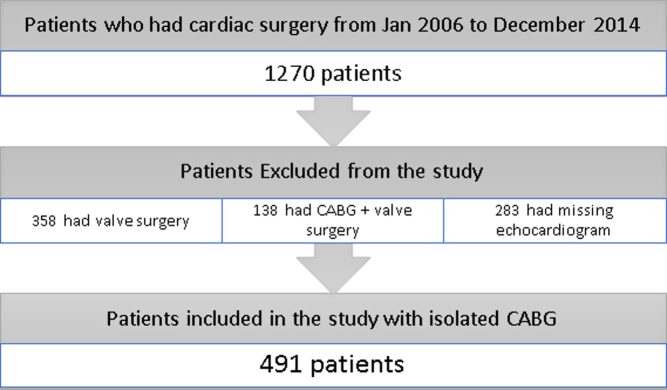
Flowchart of study sample selection based on inclusion and exclusion criteria.

Retrospective observational study was conducted at the University of Toledo Medical Center, Toledo, Ohio. The retrospective review was approved by the local audit committee. We reviewed data on all patients that underwent CABG between January 2006 and December 2014 (*n* = 1270). At our institution, patients who are at moderate-to-high risk for developing post-operative cardiac complications commonly receive a pre-operative echocardiogram. Therefore, we chose a study period of 9 years so as to allow inclusion of a reasonable number of these patients with pre-operative echocardiograms for the final analysis. Echocardiographic results were then correlated with the prospectively collected institutional cardiac surgical database as defined by the Society of Thoracic Surgeons Adult Cardiac Surgery Database. We included all patients over the age of 18 years that underwent isolated CABG (*n* = 491). Patients undergoing concomitant carotid surgery, atrial septal defect repairs or atrial fibrillation ablation procedures were excluded from the study. In order to study the impact of right-sided function on outcomes we wanted to have echocardiographic information as close to the surgery date as possible. Majority of our patients were worked up prior to surgery as outpatient and we felt that 3 months will be a reasonable period to complete pre-operative work-up including having an echocardiogram. Therefore, patients who did not have a pre-operative echocardiogram within a 3-month period prior to surgery were omitted. In addition, patients who had incomplete or missing echocardiographic data and those with both CABG and concomitant valve surgeries were also excluded from the final analysis (Fig. 1).

### Echocardiographic parameters

All pre-operative echocardiograms were performed on Phillips IE33 and GE Vivid 7 machines and analyzed with EchoPAC workstation (GE). The images were reviewed and all echocardiographic variables were remeasured by two readers who were both formally trained in echocardiography (M A C, J C). To reduce bias, both readers were blinded to the post-operative outcomes during the review process. The echocardiographic measurement protocol and reference cutoffs were based on recommendations by the American Society of Echocardiography (13, 14, 15, 16).

**Figure 2:**
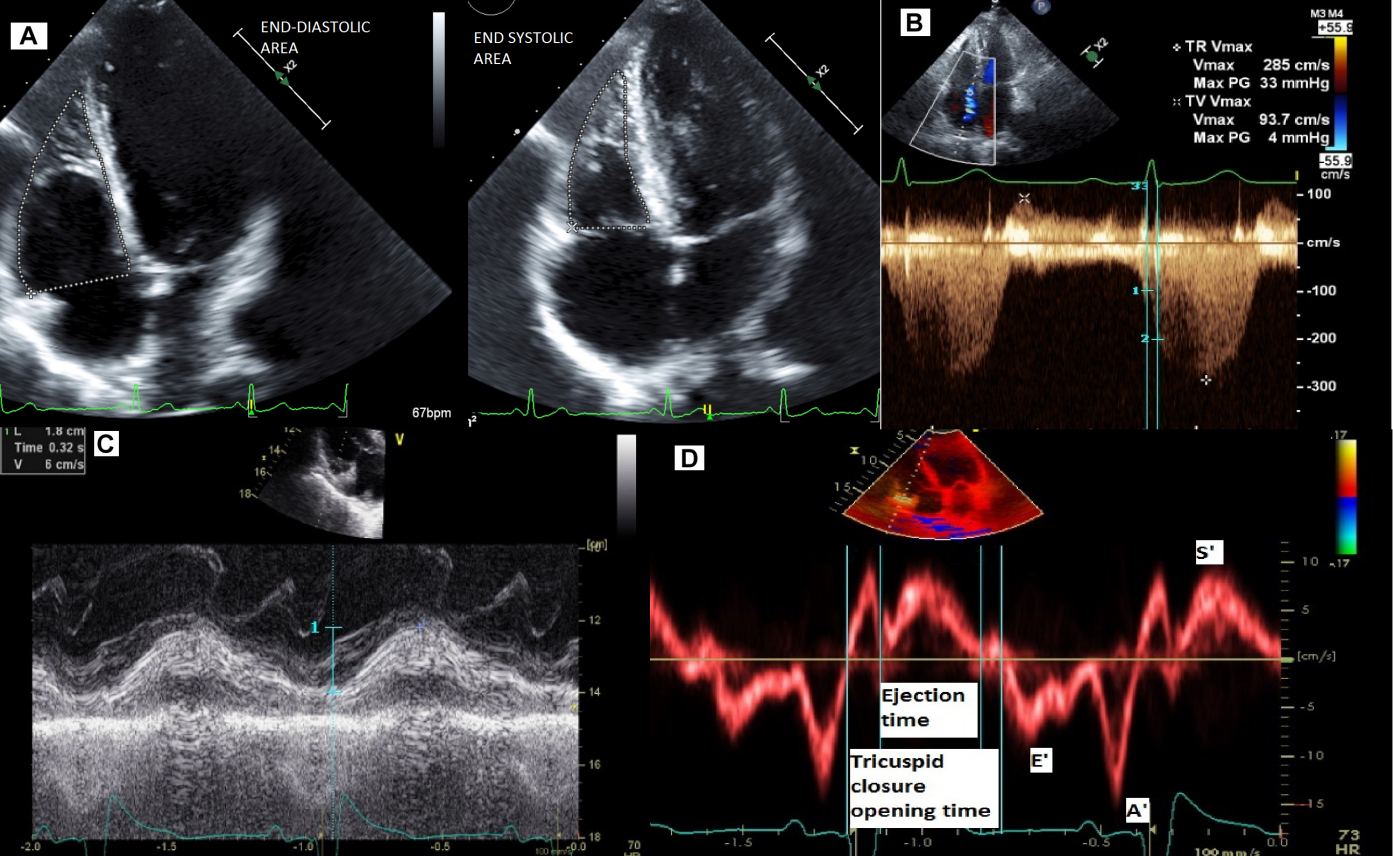
Echocardiographic illustration of RV echocardiographic parameters; (A) RVFAC, (B) RV dP/dt, (C) TAPSE, (D) TV e′, S′ and MPI.

LV ejection fraction and LV volume were measured by the biplane method of disks (modified Simpson’s rule) from the apical two- and four-chamber views. The left volume was indexed to the patient’s body surface area. Reduced LV function defined as LVEF <40% and enlarged LV volume defined as LVEDV >74mL/m^2^ for male and >61 mL/m^2^ for female and LVESV >31 mL/m^2^ for male and >24 mL/m^2^ for female. LV left atrial volume was measured by the biplane area length method indexed to patient’s body surface area. Moderately enlarged left atrium was our abnormal cutoff defined as LAVI ≥42 mL/m^2^. Mitral peak E and A wave velocities were measured from the apical four-chamber view by placing the pulsed-wave Doppler sample volume between the mitral leaflet tips while pulsed-wave tissue Doppler imaging (TDI) e′ velocity was obtained by placing the pulsed-wave Doppler sample volume at the lateral mitral basal region. Grade III diastolic dysfunction was defined as E/A >2 and increased left atrial pressure was suggested by E/e′ >14. Right atrial area was obtained by planimetry in the apical four-chamber view at the end systole. Enlarged right atrial size was defined as RA area >18 cm^2^. Basal RV diameter was measured in the basal one-third of RV inflow at end diastole in the RV focused view. RV enlargement defined by basal RV diameter >4.2 cm. Right ventricular fractional area change (RVFAC) was measured by tracing the RV border in the RV focused view at end diastole and end systole (Fig. 2A) and using the formula 100 × end diastolic area − end systolic area/end diastolic area, abnormal cutoff was defined as RVFAC <35%. RV function was also assessed visually and classified as normal or mildly, moderately or severely reduced. Moderately reduced RV function was defined by RVEF <45%. RV dP/dt was assessed from the ascending limb of the tricuspid regurgitation continuous wave Doppler signal. The time interval between 1 and 2 m/s (Fig. 2B) was measured and RV dP/dt was estimated using the formula 12 mmHg/measured time, abnormal cutoff was defined as RV dP/dt <400 mmHg/s. Abnormal tricuspid regurgitation was defined as having more than moderate tricuspid regurgitation. Tricuspid annular plane systolic excursion (TAPSE) was measured by placing the m-mode cursor through the tricuspid annulus and measuring the longitudinal excursion between end diastole and peak systole (Fig. 2C), abnormal cutoff defined as TAPSE <16 mm. Tricuspid peak E and A wave velocities were recorded in the apical four-chamber view by placing the pulsed-wave Doppler sample volume between the tricuspid leaflet tips. Tricuspid pulsed wave TDI e′, S′ velocity and RV MPI was obtained by aligning the pulsed wave Doppler sample volume with the basal RV free wall at the tricuspid annulus (Fig. 2D) and RVMPI was obtained by using the formula tricuspid valve closure opening time − ejection time/ejection time. RV diastolic dysfunction was suggested by TV E/A >2.1, elevated right atrial pressures were suggested by TV E/e′ >6, abnormal tricuspid TDI S′ was defined as <10 cm/s and abnormal RVMPI was defined as >0.55. TR peak velocity was used to estimate the maximal systolic difference between RV and right atrial pressures. Right atrial pressure was estimated from the inferior vena cava diameter and its change in diameter with respiration, abnormal cutoff defined as right atrial pressure >10 mmHg. Right ventricular systolic pressure (RVSP) was estimated by adding the right atrial pressure to the RV and right atrial systolic pressure difference. In the absence of gradient between the pulmonic valve and the right ventricular outflow tract, the RVSP was assumed to be equivalent to the systolic pulmonary artery pressure. Elevated RVSP or systolic pulmonary artery pressure was defined as more than 30 mmHg. The cutoff for PH was SPAP ≥36 mmHg.

We had a total of 16 patients (3%) who had atrial fibrillation during the time of their echocardiogram. In this group, both mitral E/a and tricuspid E/a were kept as missing variables in the final analysis due to the absence of the ‘a wave’. We also chose to use the tissue Doppler method for estimating RV MPI rather than the pulsed Doppler method in order to avoid matching the R–R intervals of the analyzed beats.

### Outcome variables

Composite outcome (POAO) was defined as occurrence of any one of the following adverse events within 30 days of CABG; prolonged mechanical ventilation duration of more than >48 h, re-intubation, intensive care unit readmission, post-operative atrial fibrillation, myocardial infarction, renal failure, stroke, cardiopulmonary arrest, multi-system failure/shock, cardiac-related hospital readmission for either congestive heart failure, myocardial infarction or arrhythmia and death. Long-term mortality was defined as death due to any cause starting from the time of surgery to three and half years post CABG. According to literature the mortality rate post CABG is highest in the first year (3.2%) which then decreases by the third year (0.9%) (17). Thus, we felt three and a half years would be a reasonable follow-up period for long-term mortality. Date of death was obtained from social security death master file and our medical records.

### Statistical analysis

Continuous variables are reported as mean ± standard deviation and categorical variables are reported as count (percent). Two sample *t* tests were used to assess differences in continuous variables and discrete variables were assessed with Fisher’s exact tests or chi-square test as appropriate. Simple correlations between left- and right-sided echocardiographic parameters with outcomes were conducted. Correlations were expressed with *r*+ correlation coefficient and *P* values were placed. *P* value of <0.05 (two sided) was considered statistically significant. Binary logistic regression analysis, multinomial logistic regression and backward elimination stepwise regression were used to assess the association of each of the echocardiographic parameters with 30-day adverse outcomes after CABG.

To assess intra-observer agreement, 29 patients with good echocardiographic images were randomly selected and the right-sided parameters were remeasured by the first reader (MAC). Inter-observer agreement was assessed by repeating the RV measurements from those same patients by a second reader (J C) who was blinded to previous measurements. Bland–Altman analysis and intraclass correlation (ICC) was used to assess intra-observer and inter-observer variability.

A Kaplan–Meier method was performed to estimate the cumulative survival. A comparative log rank test was used to compare the survival rates between different subgroup. IBM SPSS Statistics for Windows (version 22.0. Armonk, NY: IBM Corp) was used to conduct the statistical analysis.

## Results

### Baseline characteristics and descriptive statistics

A total of 774 patients underwent isolated CABG at our institute from January 2006 to December 2014. After review of the inclusion and exclusion criteria, a total of 491 patients (63%) were included in the final analysis. The average age of patient population was 66 ± 11.5 years, majority of patients were male (*n* = 338, 69%) with an average BMI of 30.5 ± 6.1 kg/m^2^ and the mean LV ejection fraction was 50.7% ± 12.6. Baseline clinical characteristics and echocardiographic variables have been outlined in Table 1.

**Table 1:** Baseline clinical characteristics and echocardiographic variables.

Baseline clinical characteristics	All patients (*n* = 491)	30-day post-operative adverse outcome		*P* value
		Yes (*n* = 227)	No (*n* = 264)	
Age (mean ± s.d.) (years)	66 ± 11.5	69 ± 11	64 ± 11.5	**0.00**
Male, no. (%)	338 (69)	149 (44.1)	189 (55.9)	0.177
Women, no. (%)	153 (31)	77 (50.3)	76 (49.7)	
BMI (mean ± s.d.) (kg/m^2^)	30.5 ± 6.1	29.9 ± 6.5	31.1 ± 5.8	**0.03**
Diabetes mellitus, no. (%)	222 (45)	105 (47.3)	117 (52.7)	0.652
Dyslipidemia, no. (%)	394 (80)	186 (47.2)	208 (52.8)	0.347
Hypertension, no. (%)	423 (86)	199 (47.0)	224 (53.0)	0.321
Chronic lung disease, no. (%)	126 (25.6)	59 (46.8)	67 (53.2)	0.867
Peripheral arterial disease, no. (%)	127 (25.8)	62 (48.8)	65 (51.2)	0.49
End stage renal disease on dialysis, no. (%)	32 (6.5)	17 (53.1)	15 (46.9)	0.33
Heart failure admission 2 weeks prior to procedure, no. (%)	110 (22)	63 (57.3)	47 (42.7)	**0.008**
NYHA III, no. (%)	58 (11.8)	34 (58.6)	24 (41.4)	**0.044**
NYHA IV, no. (%)	26 (5.3)	13 (50.0)	13 (50.0)	0.692
History of prior myocardial infarction, no. (%)	295 (60)	140 (47.5)	155 (52.5)	0.504
Previous percutaneous coronary intervention, no. (%)	135 (27.4)	62 (45.9)	73 (54.1)	0.93
Previous CABG, no. (%)	16 (3.3)	11 (68.8)	5 (31.3)	0.066
History of cardiac arrhythmia, no. (%)	102 (20.7)	51 (50.0)	51 (50.0)	0.39
Single or two vessel CABG, no. (%)	353 (71.9)	140 (39.7)	213 (60.3)	**0.00**
Multi-vessel CABG (≥3 vessels), no. (%)	138 (28.1)	87 (63)	51 (37)	
Patients who underwent cardiopulmonary bypass during CABG, no. (%)	427 (87)	196 (45.9)	231 (54.9)	0.7
Patients who underwent off pump bypass surgery, no. (%)	64 (13)	31 (48.4)	33 (51.6)	
STS predicted morbidity and mortality score, (mean ± s.d.) (%)	17 ± 12.8	20.5 ± 14	14.5 ± 10.7	**0.000**
Ejection fraction (mean ± s.d.) (%)	50.7 ± 12.6	49.5 ± 12.8	51.8 ± 12.4	0.052
Left atrial volume index (mean ± s.d.) (mL/m^2^)	27.9 ± 11.5	30 ± 13	26.2 ± 9.7	**0.001**
Left ventricular end diastolic volume (mean ± s.d.) (mL/m^2^)	60.3 ± 21.5	60.6 ± 21.8	60 ± 21.3	0.79
Left ventricular end systolic volume (mean ± s.d.) (mL/m^2^)	31 ± 17	30.6 ± 17	30.7 ± 17	0.94
E/a (mean ± s.d.)	1.2 ± 0.7	1.27 ± 0.7	1.15 ± 0.6	0.06
E/e′ (mean ± s.d.)	11.2 ± 5.5	12.1 ± 6	10 ± 5	**0.001**
Right atrial area (mean ± s.d.) (cm^2^)	14.8 ± 4.4	15.4 ± 4.6	14.3 ± 4	**0.009**
Basal right ventricle diameter (mean ± s.d.) (cm)	3.4 ± 0.6	3.4 ± 0.6	3.4 ± 0.6	0.768
Right ventricle fractional area change (mean ± s.d.) (%)	46.2 ± 12.4	45.8 ± 11.8	46.5 ± 12.8	0.555
Moderate to severely reduced RV function by visual assessment, no. (%)	31 (6)	22 (71)	9 (29)	0.052
dP/dt (mean ± s.d.) (mmHg/s)	577.9 ± 417.7	574 ± 394.4	581.9 ± 442	0.875
TAPSE (mean ± s.d.) (mm)	2 ± 0.54	1.4 ± 0.3	2.2 ± 0.4	**0.00**
TDI S′ (mean ± s.d.) (cm/s)	13.4 ± 3.7	13.4 ± 4	13.5 ± 3.6	0.796
TV E/a (mean ± s.d.)	1.22 ± 0.6	1.2 ± 0.6	1.3 ± 0.5	0.176
TV E/e′ (mean ± s.d.)	6.3 ± 3	6.24 ± 3.4	6.28 ± 2.8	0.895
Right atrial pressure (mean ± s.d.) (mmHg)	6.6 ± 3.9	6.5 ± 3.8	6.7 ± 4	0.683
RVSP (mean ± s.d.) (mmHg)	29 ± 15.4	31.6 ± 15.6	27.4 ± 15	**0.002**
RVSP >36 mmHg (possible PH), no. (%)	146 (30)	81 (55.5)	65 (44.5)	**0.008**
MPI (mean ± s.d.)	0.5 ± 0.17	0.53 ± 0.16	0.49 ± 0.16	**0.037**

Bold indicates statistical significance.

### Post-operative adverse outcomes

A total of 227 patients had POAO (46%). Post-operative atrial fibrillation was the most common POAO followed by prolonged ventilation duration, 27.3 and 12.7% respectively (Table 2). Patients who had POAO within 30 days were found to be older, had relatively lower BMI, had heart failure exacerbation requiring admission about 2 weeks prior to the procedure, were at NYHA functional class III, had multi-vessel CABG and a higher Society of Thoracic Surgeons predicted morbidity and mortality score (STS PMMS). In terms of echocardiographic variables, this group of patients were found to have increased left atrial size, left atrial pressures, right atrial size, RVSP and decreased TAPSE. They were also found to have an increased RV MPI, which is an indicator of decreased global RV function (Table 1). On univariate analysis, LVEF <40%, left atrial volume index >42 mL/m^2^, mitral E/A >2, mitral E/e′ >14, right atrial area >18 cm^2^, RVSP >30 mmHg, PH (RVSP >36 mmHg) and RV MPI >0.55 were associated with 30-day POAO. After adjusting for STS PMMS, left atrial volume index ≥42 mL/m^2^ (LAVI) (OR (95% CI): 1.98 (1.03–3.82), *P* = 0.04), mitral E/A >2 (1.97 (1.02–3.78), *P* = 0.04), right atrial size >18 cm^2^ (1.86 (1.14–3.05), *P* = 0.01), TAPSE <16 mm (1.8 (1.03–3.17), *P* = 0.04), right ventricular systolic pressure (RVSP) ≥30 mmHg (1.66 (1.14–2.43), *P* = 0.01), PH (RVSP >36 mmHg) (1.6 (1.03–2.38), *P* = 0.04) and RVMPI >0.55 (1.58 (1.01–2.46), *P* = 0.04) were found to be associated with 30-day POAO (Table 3).

**Table 2:** Post-operative adverse outcomes within 30 days of CABG.

30-day post CABG outcomes	All patients (*n* = 491)
Prolonged ventilation duration (>24 h), no. (%)	63 (12.8)
Patients requiring re-intubation, no. (%)	36 (7.3)
Intensive care unit readmission, no. (%)	16 (3.3)
Post-operative stroke, no. (%)	8 (1.6)
Post-operative renal failure, no. (%)	21 (4.3)
Cardiopulmonary arrest, no. (%)	13 (2.6)
Multi-system failure/shock, no. (%)	9 (1.8)
Post-operative atrial fibrillation, no. (%)	134 (27.3)
Post-operative myocardial infarction, no. (%)	15 (3)
Mortality within 30 days of CABG, no. (%)	21 (4.3)
Cardiac related hospital readmission (congestive heart failure, myocardial infarction or arrhythmia), no. (%)	45 (9.1)

**Table 3:** Echocardiographic variables and their association with 30 days post CABG composite adverse outcomes.

Variable	Unadjusted			Adjusted		
	*P* value	Odds ratio	Confidence interval	*P* value	Odds ratio	Confidence interval
Ejection fraction <40%	**0.04**	**1.59**	**1.03–2.46**	0.23	1.33	0.83–2.12
Left atrial volume index ≥42 mL/m^2^	**0.02**	**2.08**	**1.15–3.76**	**0.04**	**1.98**	**1.03–3.82**
Left ventricular end diastolic volume normalized by BSA >74 mL/m^2^ for male and >61 mL/m^2^ for female	0.12	1.38	0.92–2.07	0.17	1.34	0.88–2.05
Left ventricular end systolic volume normalized by BSA >31 mL/m^2^ for male and >24 mL/m^2^ for female	0.41	1.17	0.8–1.71	0.59	1.12	0.75–1.66
Mitral E/A >2	**0.03**	**1.92**	**1.05–3.5**	**0.04**	**1.97**	**1.02–3.78**
Mitral E/e′ >14	**0.00**	**1.99**	**1.3–3.04**	0.07	1.52	0.96–2.39
Right atrial size >18 cm^2^	**0.01**	**1.85**	**1.17–2.92**	**0.01**	**1.86**	**1.14–3.05**
Right ventricle basal diameter >4.2 cm	0.59	1.16	0.67–2.01	0.72	1.11	0.63–2
Right ventricle fractional area change <35%	0.55	1.16	0.71–1.9	0.89	1.04	0.62–1.74
Visual assessment of right ventricle ejection fraction <45%	0.06	0.46	0.21–1.02	0.07	0.47	0.2–1.1
Right ventricle dP/dt <400 mmHg/s	0.44	0.83	0.53–1.33	0.23	0.73	0.44–1.22
Severity of tricuspid regurgitation (more than moderate TR)	0.26	1.39	0.79–2.45	0.27	1.4	0.77–2.56
TAPSE <16 mm	**0.01**	**2**	**1.2–3.35**	**0.04**	**1.8**	**1.03–3.17**
Tricuspid tissue Doppler S′ <10 cm/s	0.83	0.94	0.52–1.7	0.52	0.81	0.42–1.57
Tricuspid E/A >2.1	0.3	0.53	0.16–1.74	0.57	0.7	0.2–2.4
Tricuspid E/e′ >6	0.53	0.88	0.59–1.32	0.86	0.96	0.63–1.48
Right atrial pressure >10 mmHg	0.89	0.97	0.59–1.6	0.28	0.74	0.43–1.27
Right ventricular systolic pressure >30 mmHg	**0.00**	**1.77**	**1.24–2.54**	**0.01**	**1.66**	**1.14–2.43**
Pulmonary hypertension (RVSP >36 mmHg)	**0.00**	**1.9**	**1.27–2.75**	**0.04**	**1.6**	**1.03–2.38**
Right myocardial performance index >0.55	**0.04**	**1.56**	**1.03–2.37**	**0.04**	**1.58**	**1.01–2.46**

E/e′ ≥14.

Bold indicates statistical significance.

We also investigated associations between all the individual POAO and the echocardiographic variables, we found the following: MPI >0.55 (2.1 (1.07–4.06), *P* = 0.03), RVSP ≥36 mmHg (PH) (1.8 (1.04–3.06), *P* = 0.03) and mitral valve E/e′ >14 (2.78 (1.58–4.79) *P* = 0.00) were associated with prolonged ventilation duration (>48 h), while PH was found to be associated with the development of post-operative atrial fibrillation (1.8 (1.2–2.70) *P* = 0.01).

Out of the 227 patients with 30-day POAO, 80 patients (35%) had more than one post-operative event within the 30-day period. Increased left atrial pressure (*r* = 0.182, *P* = 0.00), increased right atrial size (*r* = 0.108, *P* = 0.019) and RVSP ≥36 mmHg (PH) (*r* = 0.124, *P* = 0.01) correlated with having more than one adverse event within the 30-day period.

### Ejection fraction

To study the impact of ejection fraction and RVD on POAO, we divided the study population into two groups: LVEF ≥40% (*n* = 388, 79%) and LVEF ≤39% (*n* = 103, 21%). In patients with LVEF >40%, LAVI >42 mL/m^2^ (*P* = 0.002, CI: 1.5–6.6), E/e′ >14 (*P* = 0.003, CI: 1.3–3.7) and MPI >0.54 (*P* = 0.044, CI: 1.01–2.68) were associated with 30-day POAO. While in patients with reduced LV function none of the echocardiographic parameters were seen to have any association with 30-day POAO. The comparatively lesser number of subjects in the LVEF ≤39% group could be a possible reason for not finding any associations between RV echocardiographic indices and 30-days POAO.

### CABG

Overall, 71.7% (*n* = 352) of patients had single- or two-vessel CABG while 28.1% (*n* = 139) had multi-vessel CABG. Patients with multi-vessel CABG were found to have more POAO compared to single- or two-vessel CABG (odds ratio: 0.61, CI: 0.4–0.9, *P* = 0.014). No relationship between number of grafts and long-term mortality was observed.

A total of 427 patients out of 491 (87%) patients underwent cardiopulmonary bypass during surgery while the remaining patients had off pump bypass surgery. No significant difference in the incidence of POAO was observed in patients who underwent cardiopulmonary bypass vs those who underwent off pump bypass surgery (Table 1).

### Relationship between left and RV

**Figure 3:**
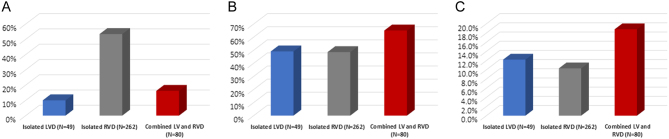
Graphs showing percentage of (A) isolated left ventricular dysfunction (LVD), isolated right ventricular dysfunction (RVD) and combined left and right ventricular dysfunction (LV and RVD), (B) incidence of POAO in the three groups, (C) incidence of long-term mortality in the three groups.

LV ejection fraction correlated with MPI (*r* = −0.129, *P* = 0.12), RVSP (*r* = −0.211, *P* = 0.00), TAPSE (*r* = 0.184, *P* = 0.001), RVFAC (*r* = 0.136, *P* = 0.005), RV diameter (*r* = −0.213, *P* = 0.00), right atrial area (*r* = −0.191, *P* = 0.00), mitral E/a (*r* = −0.276, *P* = 0.00), mitral E/e′ (*r* = −0.252, *P* = 0.00) and left atrial size (*r* = −0.212, *P* = 0.00). Showing that decreasing LV function results in LV diastolic dysfunction, increased left atrial pressure, increased RVSP, enlarged right atrium and RV dysfunction. While left atrial pressure (E/e′) correlated with RVSP (*r* = 0.332, *P* = 0.00), right atrial pressure (*r* = 0.154, *P* = 0.001), right atrial size (*r* = 0.121, *P* = 0.12), TAPSE (*r* = −0.116, *P* = 0.38) and dP/dt (*r* = −0.131, *P* = 0.026). Showing that increasing left atrial pressure increases RVSP, right atrial pressure and size and decreases RV systolic function. Left ventricular dysfunction (LVD) was defined as having an ejection fraction less than 40% and 49 out of 491 patients had isolated LVD (10%). Right ventricular dysfunction (RVD) was defined as having an abnormal RV echocardiographic parameter; right atrial size >18 cm^2^, TAPSE <16 mm, RVSP ≥30 mmHg or RVMPI >0.55 (Table 3) and 262 out of 491 patients had isolated RVD (53.4%). While 80 patients out of 491 had combined LV and RVD (16.2%) (Fig. 3A), POAO was observed in 65% (*n* = 52/80) of patients with both LV and RVD, 49% (24/49) of patients with isolated LVD and 48.5% (*n* = 127/262) of patients with isolated RVD (Fig. 3B). When looking at long-term mortality, 18.8% (*n* = 15/80) patients with both LV and RVD, 12.2% (*n* = 6/49) with isolated LVD and 10.3% (*n* = 27/262) with isolated RVD expired on long-term follow-up (Fig. 3C).

Combined LV and RVD was found to be significantly associated with POAO (OR: 2.5, *P* = 0.00, CI: 1.5–4) and long-term mortality (hazard ratio 1.9, *P* = 0.04, CI: 1.03–3.7). While both isolated LV and RVD had similar incidences of short- and long-term adverse outcomes. There were lesser number of patients in the isolated LVD group compared to isolated RVD group (49 vs 262) suggesting that the incidence of POAO and long-term mortality is probably increased in isolated LV compared to isolated RV. However, these observations were not statistically significant.

### Post-operative statistics

Another aim of the study was to look at echocardiogram within 1 month of CABG to see if patients with pre-operative RV dysfunction developed POAO and long-term mortality due to worsening of their RV echocardiographic indices or RV function. However, when we retrospectively looked at post-operative echocardiogram, we found that majority of our patients did not have a follow-up echocardiogram within a month of CABG since they had no change in clinical status and did not fulfill the appropriate criteria for echocardiography (18). Therefore, we decided to change our protocol and include all patients with post-operative echocardiogram within 2 years of CABG for a subgroup analysis. We intended to study what kind of impact CABG had on long-term RV function – did it improve or get worse with time post CABG. As outlined in Table 3, we decided to analyze the variables which were associated with POAO in our pre-CABG analysis – LVEF, left atrial size, right atrial size, mitral E/A, mitral E/e′, RVSP, TAPSE and RVMPI.

Only 169 patients (34%) had post CABG echocardiogram on follow-up within a median of 2.5 (IQR: 1–12) months from the procedure. We looked at the indication for the post-procedure echocardiogram and the major reasons were the following: chest pain (*N* = 20, 12%), shortness of breath (*N* = 17, 10%) and congestive heart failure exacerbation (*N* = 13, 7.7%). While the remaining 70% were due to miscellaneous reasons including follow-up echocardiogram for valvular disease, PH, rule out pericardial effusion, syncope and pre-syncopal episodes, arrythmias and post-CABG cardiac arrest. Following CABG, majority of patients had no change in their LVEF (*n* = 77, 46%) and TAPSE (*n* = 118, 70%), had an improvement in left atrial pressures (*n* = 90, 53%) but had worsening of their left atrial size (*n* = 99, 59%), right atrial size (*n* = 111, 66%), RVSP (*n* = 86, 51%) and RVMPI (*n* = 100, 59%). Both the left and right atria showed significant changes in size post CABG. Mean pre-operative left atrium volume index was 28 ± 12 mL/m^2^, which increased post CABG to 31 ± 14 mL/m^2^ (*P* = 0.038). Similarly, mean pre-operative right atrial area was 29 ± 16 cm^2^ which increased post CABG to 32 ± 15 cm^2^ (*P* = 0.00). Therefore, both the left and the right atrium undergo remodeling post CABG. Percentage change in pre and post CABG echocardiographic variables did not show any association with POAO or long-term mortality. Post CABG, the following persistently abnormal echocardiographic parameters were found to be associated with 30-day POAO; right atrial size >18 cm^2^ (2.77 (1.26–6.08), *P* = 0.01), RVSP >30 mmHg (2.32 (1.25–4.29), *P* = 0.01) and MPI >0.55 (3.29 (1.64–6.61), *P* = 0.00). Interestingly we can conclude that persistent RVD which does not improve following CABG could potentially be a marker for 30-day POAO. Unfortunately, we did not have sufficient data to study the association between persistent RVD post CABG and long-term mortality.

### Short-term and long-term mortality

**Figure 4:**
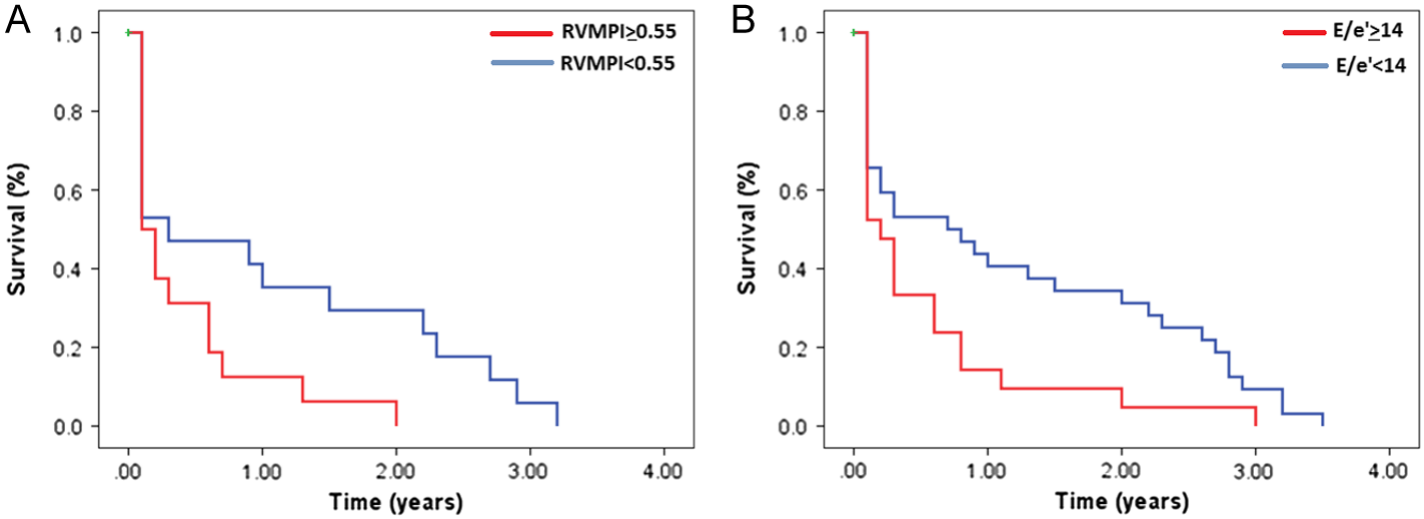
Cumulative survival over three and half years follow-up period. (A) RVMPI ≥0.54, (B) mitral valve E/e′ ≥14.

A total of 21 patients expired within 1 month post CABG (4.3%). Within the 30 days following CABG, nine patients had multisystem failure out of which seven died (78%) and 13 patients had cardiopulmonary arrest out of which seven died (54%). Thirty-day mortality was found to be associated with increased left atrial pressures as measured by mitral E/e′ >14 (2.9 (1.15–7.1), *P* = 0.02). Long-term mortality included all patients who died within 30 days and also those who died within three and half years after CABG. A total of 53 patients died over the follow-up period (11%). Cumulative survival was decreased in patients with RVMPI ≥0.55 (log rank: 4.5, *P* = 0.034) and in patients with mitral valve E/e′ ≥14 (log rank: 4.9, *P* = 0.026) (Fig. 4).

### Interrater variability

Bland–Altman analysis of intra-observer variability of the RV echocardiographic parameters showed the following: right atrial size, 0.09 ± 0.98 cm^2^; basal RV diameter, −0.08 ± 0.228 cm; RVFAC, 1.7 ± 9.2%; TAPSE, 0.07 ± 0.2 cm; TDI S′, 0.05 ± 0.22 cm/s; tricuspid valve E/e′, −0.09 ± 0.59; RVSP, 1 ± 3 and RVMPI, 0.01 ± 0.04. ICC for the variables were the following: right atrial size, 0.98; basal RV diameter, 0.95; RVFAC, 0.89; TAPSE, 0.95; TDI S′, 0.99; tricuspid valve E/e′, 0.98; RVSP, 0.99 and RVMPI, 0.98.

Bland–Altman analysis of inter-observer variability of the RV echocardiographic parameters showed the following: right atrial size, −0.38 ± 2.39; basal RV diameter, −0.035 ± 0.42, RVFAC, 4.85 ± 10.3; TAPSE, 0.01 ± 0.24; TDI S′, 0.1 ± 2.29; tricuspid valve E/e′, 0.26 ± 1.3; RVSP, 4.44 ± 8.59 and RVMPI, −0.01 ± 0.1. ICC for the variables were the following: right atrial size, 0.88; basal RV diameter, 0.88; RVFAC, 0.84; TAPSE, 0.92; TDI S′, 0.9; tricuspid valve E/e′, 0.93; RVSP, 0.94 and RVMPI, 0.835.

A value between 0.75 and 0.9 is an indicator of good reliability, whereas a value more than 0.9 is an indicator of excellent reliability (19). In the intra-observer group, all the RV echocardiographic indices showed excellent reliability apart from RVFAC which showed good correlation. Whereas in the inter-observer group RVSP, TV E/e′, TDI S′ and TAPSE showed excellent correlation while right atrial size, basal RV diameter, RVFAC and RVMPI showed good correlation.

## Discussion

Our study shows the importance of pre-operative echocardiography as a screening modality for patients scheduled for CABG and highlights the role of RV and its relationship with short-term and long-term post-surgical outcomes. Increased left and right atrial size, grade III diastolic dysfunction, TAPSE, PH and abnormal RVMPI were found to be strongly associated with 30-days POAO while RVMPI ≥0.55 and mitral valve E/e′ ≥14 were associated with long-term mortality.

Reduced right ventricular function following myocardial infarction is a major risk factor for death, heart failure, stroke (20) and long-term mortality (21). Following myocardial infarction, RVD develops due to RV ischemia or as a consequence of left heart disease. LV failure increases pulmonary venous and the pulmonary arterial pressure, thus increasing RV afterload and right-sided workload. In addition, LV dysfunction could decrease RV coronary perfusion pressures leading to RV ischemia and further injury. LV dilation via ventricular interdependence could also restrict RV diastolic filling (22). Therefore, LV function post myocardial infarction can influence the RV and as demonstrated by our findings, having combined LV and RVD significantly increases the risk for POAO and long-term mortality.

Pre-operative RVD is a predictor of POAO following CABG (23, 24). The culprits of post-operative RV failure are sudden increase in pulmonary arterial pressures and RV ischemia. Acute-onset PH can be due to pulmonary vasoconstriction after removal of cardiopulmonary bypass, ischemia reperfusion syndrome, reaction to protamine, transfusions or metabolic abnormalities. RV ischemia can be caused by air embolism, thromboembolic events, graft dysfunction or hypotension (25). A normal RV might be able to tolerate the new-onset PH and RVD. However, patients with pre-operative RVD are often not able to endure these sudden changes and develop RV insufficiency, which leads to a low cardiac output syndrome. The failing RV will affect LV function by ventricular interdependence and decrease the LV preload, eventually resulting in circulatory collapse (22, 25).

RV MPI globally estimates both the systolic and diastolic function of the RV. Its main advantage being that it is independent of geometric assumptions while being relatively independent of preload and afterload (15). The importance of RV MPI as a predictor of post-operative mortality and morbidity in patients undergoing cardiac surgery has previously been reported (10, 23, 26). In our study, MPI was found to be associated with POAO, prolonged mechanical ventilation duration and long-term mortality. Similarly, TAPSE has been established to have prognostic value in patients with cardiomyopathy (27). In our study, TAPSE <16 mm was found to be also associated with POAO. However, we did not find any association between POAO and other systolic RV echocardiographic indices such as RVFAC and TDI S′. Reduced RVFAC has been established as a predictor of POAO and decreased survival in heart failure patients (20, 24, 28, 29). The average cutoff for LVEF in those studies were below 35%. Likewise, tricuspid TDI S′ (30) has also been reported to be predictor of adverse outcomes in patients with LVEF <35%. Our study had comparatively a smaller sample of patients with LVEF ≤35% (*n* = 71, 14%) and advanced heart failure. This could well be the reason why we did not find any association between POAO and RVFAC or TDI S′. Therefore, MPI which takes into account both the RV systolic and diastolic functional status and TAPSE are relatively better in detecting subclinical RVD as demonstrated by our findings.

Increased left atrial size is not only a surrogate marker for LV diastolic dysfunction and chronically elevated left atrial pressures but also for RVD. Increased left atrial pressures over time increases pulmonary venous pressures and eventually affects RV function. After myocardial infarction, the scared myocardium affects LV relaxation and filling pressures and with time eventually results in left atrial dilation. Adverse LA remodeling following STEMI has been reported to be associated with unfavorable prognosis (31). Similarly, increased right atrial size is the result of remodeling due to RVD and increase right-sided pressures. In our study both increased left and right atrial size were found to be associated with 30-day POAO. In addition, increased right atrial size was associated with having more than one adverse outcome within 30 days of CABG. Thus, atrial enlargement represents chronic ventricular dysfunction and can be used as a marker to anticipate short-term post-operative adverse outcomes.

Diastolic dysfunction with elevated LV end diastolic pressure has been reported to increase perioperative mortality and morbidity (32). In our study, grade III diastolic dysfunction was associated with 30-day POAO while increased left atrial pressures (mitral E/e′ >14) were associated with prolonged mechanical ventilation duration, death within 30 days of CABG and having more than one adverse outcome within 30 days of CABG. One possible explanation could be that the changes in pulmonary pressures during CABG add onto the pre-existing elevated left atrial pressures which cumulatively increase the RV workload and result in RV failure and circulatory collapse. This finding highlights the significance of increased left atrial pressures and the importance of volume status optimization prior to surgery.

The impact of PH on patients undergoing cardiac valve surgeries has already been established (12, 33, 34), and it is one of the variables used by EURSCORE to calculate post-operative risks (35). Similarly, in our study, PH was associated with 30-day POAO and prolonged mechanical ventilation duration. In addition, patients with PH usually had more than one complication during the 30 days post-operative period. Therefore, patients with ischemic heart disease who develop PH are at increased risk for POAO and RVSP ≥36 mmHg when detected on echocardiogram will warrant further invasive workup and management in order to improve post-operative outcomes.

On long-term follow up, patients with pre-operative RV MPI >0.55 (RVD) or mitral valve E/e′ >14 (increased left atrial pressures) had increased all-cause mortality at the end of three and half years. This finding highlights the fact that patients who develop RVD as a result of elevated left atrial pressures and pulmonary venous hypertension are at increased risk for long-term mortality. The observed mortality trend on long-term follow-up suggests that the RV might have a larger contributory role to the overall cardiac function than previously thought. It is possible that even after CABG, in the presence of persistent LV stiffness; the RV might continue to undergo remodeling which with time eventually leads to long-term mortality. Therefore, post CABG, there might be a benefit in regularly monitoring such at-risk patient population and adequately optimizing their heart failure management.

### Limitations

The major limitation is that this is a retrospective study with a small sample size. The study was conducted in a single center and involved patients with similar ethnic characteristics. Conducting the study in two to three centers would have increased the sample size and the diversity of the patient population resulting in increased validity of the observed findings. Our study sample could potentially reflect a sicker population since moderate-to-high-risk patients are usually evaluated with pre-operative echocardiograms. This could explain the high number of POAO observed in our study. The quality of the echocardiographic images and RV functional assessment was limited in some of the patients by their body habitus. We also had a limited number of follow-up echocardiograms post CABG, which restricted our post-operative analysis. Finally, only 53 patients were found to have a mortality event on post CABG follow-up. Since this is a relatively smaller number of events, we did not pursue further multivariate survival analysis. Even though, the mortality data are small it does suggest a relationship between pre-CABG E/e′ and RVMPI with long-term mortality and larger sample sized prospective studies will help to further validate this observation.

## Conclusion

In our study, we showed the importance of echocardiography as a non-invasive screening tool for identifying patients at high risk for post-operative complications. We also demonstrated the importance of right ventricle for the overall function of the heart since pre-operative RVD was associated with both POAO and long-term mortality. In addition, we highlighted the importance of left atrial pressures and atrial size and its impact on short-term outcomes. Thus, patients identified to have increased left atrial pressures, PH or RVD prior to CABG might benefit from optimization of volume status prior to surgery in order to improve outcomes, reduce post-operative complications and medical costs. In addition, patients with RVD or increased left atrial pressures will need long-term monitoring and medication optimization in order to decrease long-term mortality.

## Declaration of interest

The authors declare that there is no conflict of interest that could be perceived as prejudicing the impartiality of the research reported.

## Funding

This work did not receive any specific grant from any funding agency in the public, commercial, or not-for-profit sector.
